# The importance of propolis in alleviating the negative physiological effects of heat stress in quail chicks

**DOI:** 10.1371/journal.pone.0186907

**Published:** 2017-10-20

**Authors:** Gamal M. K. Mehaisen, Rania M. Ibrahim, Adel A. Desoky, Hosam M. Safaa, Osama A. El-Sayed, Ahmed O. Abass

**Affiliations:** 1 Department of Animal Production, Faculty of Agriculture, Cairo University, Giza, Egypt; 2 Poultry Cellular and Molecular Physiology Laboratory, Faculty of Agriculture, Cairo University, Giza, Egypt; 3 Poultry Breeding Department, Animal Production Research Institute, Dokki, Giza, Egypt; Wageningen UR Livestock Research, NETHERLANDS

## Abstract

Heat stress is one of the most detrimental confrontations in tropical and subtropical regions of the world, causing considerable economic losses in poultry production. Propolis, a resinous product of worker honeybees, possesses several biological activities that could be used to alleviate the deleterious effects of high environmental temperature on poultry production. The current study was aimed at evaluating the effects of propolis supplementation to Japanese quail (*Coturnix coturnix japonica*) diets on the production performance, intestinal histomorphology, relative physiological and immunological parameters, and selected gene expression under heat stress conditions. Three hundred one-day-old Japanese quail chicks were randomly distributed into 20 wired-cages. At 28 d of age, the birds were divided into 2 temperature treatment groups; a normal at 24°C (C group) and a heat stress at 35°C (HS group). The birds in each group were further assigned to 2 subgroups; one of them was fed on a basal diet without propolis supplementation (-Pr subgroup) while the other was supplemented with propolis (+Pr subgroup). Production performance including body weight gain, feed intake and feed conversion ratio were measured. The intestinal histomorphological measurements were also performed for all treatment groups. Relative physiological parameters including body temperature, corticosterone hormone level, malondialdehyde (MDA) and free triiodothyronine hormone (fT3), as well as the relative immunological parameters including the total white blood cells count (TWBC’s), heterophil/lymphocyte (H/L) ratio and lymphocyte proliferation index, were also measured. Furthermore, the mRNA expression for toll like receptor 5 (TLR5), cysteine-aspartic protease-6 (CASP6) and heat shock proteins 70 and 90 (Hsp70 and Hsp90) genes was quantified in this study. The quail production performance was significantly (P<0.05) impaired by HS treatment, while Pr treatment significantly improved the quail production performance. The villus width and area were significantly (P<0.05) lower in the HS compared to the C group, while Pr treatment significantly increased crypts depth of quail. A negative impact of HS treatment was observed on the physiological status of quail; however, propolis significantly alleviated this negative effect. Moreover, quail of the HS group expressed lower immunological parameters than C group, while propolis enhanced the immune status of the quail. The relative mRNA expression of TLR5 gene was down-regulated by HS treatment while it was up-regulated by the Pr treatment. Furthermore, the positive effects of propolis in HS-quail were evidenced by normalizing the high expressions of CASP6 and Hsp70 genes when compared to the C group. Based on these results, the addition of propolis to quail diets as a potential nutritional strategy in order to improve their performance, especially under heat stress conditions, is recommended.

## Introduction

Japanese quail (*Coturnix coturnix japonica*) is considered one of the locally available and a cheap sources of poultry meat [1]. Quail meat is renowned for its dietary properties like high-quality protein and low-caloric content, and its valuable taste to consumers [2,3]. It is also a preferable experimental animal model for its unique characteristics and advantages over other species of poultry, which include early attainment of sexual maturity and short generation interval which normally produce several generations in a year [4].

Undoubtedly, global warming phenomenon is currently a serious challenge facing poultry production in tropical and subtropical regions. Heat stress begins when the ambient temperature raises the thermo-neutral zone which ranges between 16–25°C for poultry species [5]. Recent studies found that poultry flocks subjected to chronic heat stress environment (over 30°C) had poor production performance as well as high morbidity and mortality, causing substantial economic losses in poultry farms [6,7]. In response to the heat stress condition, multiple changes in poultry physiology occur in order to maintain their homeostasis status [8]. Several researchers reported reduced concentrations of thyroid hormones in heat-stressed chickens [9], affecting metabolism and growth processes in target tissues [10]. Other reports concluded that heat stress results in an irregular systemic immune and an imbalance expression of inflammatory molecules throughout the body [11]. In addition, heat stress causes the release of corticosterone [12] and initiates lipid peroxidation in cell membranes as a consequence of increased reactive oxygen species (ROS) and free radicals formation [13]. Moreover, it is indicated that exposure to acute heat stress causes a substantial impairment in the gut formation, intestinal epithelial integrity and villus morphology in laying hens [14]. Furthermore, it has been reported that poultry exposure to stress conditions, including heat stress, could have differential influences on the expression pattern of some genes such as apoptotic and heat shock proteins [15–17].

Recent studies have revealed that supplementation of some antioxidants like vitamin C and E and/or other compounds could counteract the heat stress effects on growth and physiological aspects that are caused by high environmental temperatures in broilers [18,19], in laying hens [20,21], and in quail birds [22,23]. Propolis is an adhesive resinous balsam made by worker honeybees (*Apis melifera*) [24], and it has been extensively used in poultry feeds to expand their productive performance under high environmental temperature conditions [12,19,25,26]. Studies in the last two decades explored many biological and positive characteristics for propolis such as growth promotion, appetite enhancement, antioxidation, antimicrobial, antitumor and immune modulation [27–29].

Due to this wide range of biological activities of propolis, there is a renewed interest in its use as an alternative and practical way to alleviate the deleterious effects of high environmental temperature on poultry production. However, most investigations on quail were exposed exposed to heat stress have focused on describing the positive effects of propolis on aspects correlated with productive performance while the physiological and biological reasons behind these effects remain poorly understood. Therefore, the present study was conducted to evaluate the effects of propolis supplementation into quail diets on productive performance, intestinal histomorphology, physiological aspects of inflammation, and immunological characteristics, as well as the mRNA expression of some immune-stimulatory, apoptotic and heat-shock-protein genes under heat stress conditions.

## Materials and methods

### Propolis preparation

Propolis was collected from apiary located at Faculty of Agriculture, Cairo University (Giza province, Egypt). The collected propolis was kept dried in the dark until processing according to methods described by Seven et al. [30]. Briefly, collected propolis was extracted for a week with 100 ml of 70% ethanol at room temperature. After filtration, the extract was evaporated using a vacuum evaporator at 50°C. The extracted propolis was kept in a clean, dark bottle at 4°C until use in the experiment. Propolis was incorporated into the basal diet of quail in a powder form at 1 g of extracted propolis per kg feed.

The free radical scavenging activity of extracted propolis was measured according to methods described by Oktay et al. [31]. In brief, the extracted propolis was added to a solution of 0.1 mM of 1,1-diphenyl-2-picryl-hydrazil (DPPH^•^) in methanol at different concentrations (25–75 μg/ml). The mixtures were shaken vigorously and were allowed to stand at room temperature for 30 min. Thereafter, the absorbance of reactions was measured using an automatic scanning spectrophotometer at 517 nm. The free radical scavenging activity was calculated for the extracted propolis was 87.3%.

The flavonoids and polyphenols were also measured in the extracted propolis according to methods described by Rolim et al. [32] and Kujala et al. [33], respectively. First, 1 g of extracted propolis was dissolved in 10 ml solution of ethanol 95% and glacial acetic acid 99:1 (v/v). For flavonoids assay, 1 ml of propolis solution was diluted to 100 ml with ethanol 95% and glacial acetic acid solution 99:1 (v/v) and then was measured spectrophotometrically at 363 nm. For polyphenols assay, 5 ml of propolis solution was diluted to 100 ml with ethanol then 1 ml of the diluted solution was added to 4 ml Folin-Ciocalteu reagent/water solution 1:9 (v/v). After vortex, 5 ml of sodium carbonate (7.5%) was added to the solution and left in dark for 30 min. The blue color amount that was developed from the last step was detected using automatic scanning spectrophotometer at 745 nm. As a result, the flavonoids and polyphenols amounts in the propolis samples were 10.2% and 5.6%, respectively.

### Birds and management

A total of 300, one-day-old, Japanese quail (Coturnix coturnix japonica) chicks were distributed into 20 wired-cages (15 chicks per cage, measured at 60×50×50 cm) in an environmentally-controlled room. A brooding temperature was set at 37°C on day 1 and then was gradually reduced to 24°C by d 21. Light was provided continuously (24 h) throughout the experiment. The birds were fed according to NRC (1994) guidelines with a starter diet (24% CP and 2900 kcal/kg ME) until 20 d of age followed by a grower diet (20% CP and 3100 kcal/kg ME) from d 21 onwards. The diets and fresh water were offered ad libitum.

### Experimental design and data collection

At 28 d of age, the quail were randomly distributed into two identical environmentally-controlled rooms. The two rooms were fully cleaned before initiation of the experiment and contained the same conditions of size, ventilation, humidity, light intensity, and light schedule. The temperature in the first room was kept at 24°C (C group) and the other room was kept under heat stress at 35°C (HS group). The birds in each group were further assigned to two subgroups according to dietary propolis supplementation (5 cages per subgroup), and one of them was fed on a basal diet without propolis supplementation (-Pr subgroup) while the other was supplemented with propolis (+Pr subgroup). The relative humidity of the two rooms was maintained at 50%. These treatments continued till the birds were 35 d of age.

The production performance was obtained for each treatment group as will be mentioned later. At the end of the experiment (35 d), body temperatures were measured for each treatment group and blood samples were collected in heparinized tubes for physiological and immunological analysis listed later. The intestinal histomorphological measurements were also performed for all treatment groups. In addition, the liver was separated and directly plunged into liquid nitrogen (LN_2_) for later mRNA quantification of toll-like receptor 5 (TLR5), cysteine-aspartic protease-6 (CASP6), and heat shock proteins 70 and 90 (Hsp70 and Hsp90) genes.

### Compliance with ethical standards

Birds were monitored closely twice a day to detect any signs of chronic stress (breathing difficulty, watery discharge of the peak, decreased appetite, ruffled feathers or droopy looking) throughout the experimental period. Accordingly, when one or more of these signs appeared, cervical dislocation was used to end the life of these birds. This process was accomplished to minimize suffering of birds and to allow humane endpoints. All experimental protocols were approved by Cairo University Ethics Committee for the Care and Use of Experimental Animals in Education and Scientific Research (CU-IACUC).

### The performance traits

The initial and final body weights (g) were recorded individually at the beginning and at the end of the experiment (28 and 35 d of age). The weight gain (g/bird) was determined for each treatment group. The feed intake (g/bird) was measured for each treatment group. The feed conversion ratio was calculated for each treatment group.

### Intestinal histomorphological measurements

Ten birds from each treatment group were sacrificed and the small intestine was separated from the rest of the gastrointestinal tract as a standard procedure. The intestinal length (jejunum and ileum parts) was measured for each treatment group. For histomorphological studies, intestinal samples were cut from the middle part of ileum tissues. Tissue samples were flushed and soaked in 10% neutral buffered formalin for 72 h. Samples were trimmed and processed by dehydration in alcohol, clearing in Xylene, synthetic wax infiltration and blocking out into Paraplast tissue embedding media. At least, 5 cross sections of 3–5 μm per sample with 50 μm in-between were cut by a rotatory microtome. The sections were stained with Harris Hematoxylin and Eosin as a general staining method as outlined by Suvarna et al. [34]. The villus length, villus width, villus area and crypts depth of tissue sections were examined for each treatment group at 40X magnification under light microscope equipped with HD microscopic camera and Image analysis software (Leica Microsystems, Germany).

### Physiological parameters

At the end of the experiment (35 d), the body temperatures were measured for 10 birds of each treatment group (2 birds x 5 cages) using thermocouple rectal thermometer with a 3-cm insertion probe. Blood samples were collected in heparinized tubes and centrifuged at 2000 xg for 10 min at 4°C. The plasma was separated and stored at -20°C until analysed. The plasma corticosterone concentration (5 samples per treatment group) was measured by ELISA reader (BIOTEKELX808) using corticosterone ELISA kits (MBS701668, MyBioSource Inc., San Diego, CA, USA). The free triiodothyronine concentration in plasma (fT_3_; 5 samples per treatment group) was also measured by the automated ELISA Microplate reader using enzyme immunoassay ELISA kits (EIA-10301, Chemux BioScience Inc., South San Francisco, CA, USA). The plasma malondialdehyde (MDA; 5 samples per treatment group) concentration was determined using colorimetric assay kit (AB118970, Abcam, UK), according to the manufacturer's directions.

### Immunological parameters

Five blood samples from each treatment group were collected into heparinized tubes and assigned to measure the total white blood cells (TWBC’s) and the heterophil/lymphocyte (H/L) ratio. The TWBC’s were manually determined by mixing 490 μl of brilliant cresyl blue dye with 10 μl of the whole blood sample, and then the total leukocytes were counted under a microscope at a magnification of 200X using a hemocytometer slide [35]. The H/L ratio was determined using Hema-3 stain (cat# 22–122911, Fisher scientific, USA), according to Zhang et al. [36].

The lymphocyte proliferation assay was measured in 5 blood samples in each treatment group as described in a previous work [37]. Briefly, the peripheral blood mononuclear cells (PBMCs) were isolated from blood using histopaque-1077 (Sigma Chemical Co., St. Louis, MO) and the viable lymphocytes were detected using Trypan Blue dye. Cells were plated at 5x10^5^ cells/well and incubated with Concanavalin-A (Con-A, Sigma Chemical Co., St. Louis, MO) at 41°C in a humidified atmosphere of 5% CO_2_ for 68 h. Thereafter, cells were incubated with 3-[4,5-dimethylthiazol]-2,5-diphenyltetrazolium bromide (MTT) for further 4 h, followed by addition of 10% sodium dodecyl sulfates dissolved in 0.04 M HCl solution to each single well. Subsequently, the optical density at 570 nm was obtained using an automated ELISA reader (model 550 Microplate Reader, Bio-Rad) and the stimulation index (SI) of T-lymphocyte cells was calculated for each treatment group as follows: SI = OD570 _(stimulated cells)_ / OD570 _(unstimulated cells)_.

### mRNA quantification

Total RNA was extracted from liver tissues (10 samples for each treatment group) using the standard TRIzol® Reagent extraction method. Total RNA was treated with 1 U of RQ1 RNase-free DNase (Invitrogen, Germany) to digest DNA residues, and then re-suspended in diethylpyrocarbonate (DEPC)-treated water. The purity of total RNA was assessed spectrophotometrically at 260/280 nm, and the integrity of extracted RNA was determined by using 1.5% agarose gel electrophoresis. Then, total RNA was reverse-transcribed into cDNA by using RevertAidTM First Strand cDNA Synthesis Kit (MBI Fermentas, Germany) according to the manufacturer's directions. The cDNA templates were stored at -20°C for future quantitative real time-polymerase chain reaction (qRT-PCR).

The qRT-PCR of TLR5, CASP6, Hsp70 and Hsp90 genes were normalized to the main expression of β-actin gene, and transformed using the comparative cycle threshold (CT) method [38]. Sequence-specific primers for the qRT-PCR of the selected genes were designed using the primer-blast web interface (http://www.ncbi.nlm.nih.gov/tools/primer-blast/index.cgi) as shown in Table 1. To perform the qRT-PCR reactions, 5 μl of cDNA template was mixed with 12.5 μl of 1× SYBR® Premix Ex TaqTM (TaKaRa, Biotech. Co. Ltd., Germany), 0.5 μl sense primer (0.2 μM), 0.5 μl antisense primer (0.2 μM), 6.5 μl distilled water. The reaction program consisted of three thermal cycling phases. The first phase was set to 95.0°C for 3 min. The second phase consisted of 40 cycles in which each cycle was divided to 3 steps (95°C for 15 sec, 55°C for 30 sec, and 72°C for 30 sec). The last phase consisted of 71 cycles, started at 60°C and then increased by 0.5°C every 10 sec up to 95°C. Each experiment included a distilled water control. At the end of each qRT-PCR, a melting curve analysis was performed at 95°C to check the quality of the used primers.

**Table 1:** Details of primers used for real-time PCR quantitative analysis.

Gene symbol	Forward primer sequences	Reverse primer sequences	Product size (bp)
**TLR5**	TCACACTCAACTGTCCGAGC	CGGCAGATCGATGCACTTTG	176
**CASP6**	CAGGGACCGAAACGAGGAAA	ACAGCTTCACTGCCCTTCTC	271
**Hsp70**	ATGAAACTGAGTCGCTCGCA	CAGTCTGTTGCACCTTTGGC	166
**Hsp90**	GAAACACTCTGGGACGTGGT	TTCGACAGTCTCCGTCTTGC	158
**β-actin**	GGATGCAGAAGGAGATCACTG	CAAGTACTCCGTGTGGATCG	90

### Statistical analysis

All statistical analyses were performed using IBM SPSS 22.0 Software Package (IBM corp., NY, USA, 2013). Two-way ANOVA was used to to analyze the main effects of the heat stress (HS), dietary propolis supplementation (Pr) and their interactions (HSxPr) on the production performance traits, the intestinal histomorphological measurements, the relative physiological and immunological parameters, and the mRNA expression data for selected genes. When significant differences due to the HS, Pr or interaction effects were detected, a multiple pairwise comparison among treatment groups was performed using Tukey’s HSD test. The experimental unit for each test done is the number of observations taken from each treatment group. The level of statistical significance was set at P<0.05.

## Results

### The performance traits

Results of the quail production performance under heat stress with or without dietary propolis supplementation are shown in Table 2. The average body weight gain from 28–35 d of age was significantly (P<0.05) less in the HS group compared to the C group. However, dietary propolis supplementation significantly increased the body weight gain in both HS and C groups. Quail in the HS group had significantly less feed intake and worse feed conversion ratio compared to the C group. However, dietary propolis supplementation ameliorates the negative effects of HS on quail and significantly increased the feed intake and feed efficiency.

**Table 2:** Least square means for the production performance traits as affected by heat stress and dietary propolis supplementation in Japanese quail.

Items	n	Treatment groups ^1^				SEM	Main effects ^2^		
		C		HS					
		- Pr	+ Pr	- Pr	+ Pr		HS	Pr	HSxPr
Initial body weight (g)	25	192.0	193.7	190.1	189.5	2.58	NS	NS	NS
Body weight gain (g/bird)	25	29.0 ^b^	37.9 ^a^	16.0 ^d^	26.0 ^c^	0.33	S	S	NS
Feed Intake (g/bird)	5	101.0 ^b^	108.7 ^a^	65.0 ^d^	83.2 ^c^	1.32	S	S	S
Feed conversion ratio	5	3.5 ^b^	2.9 ^d^	4.1 ^a^	3.2 ^c^	0.04	S	S	S

^a-d^ Means with different superscripts, within the same row, are significantly different (P<0.05).

n: number of observations per treatment group. SEM: standard error of the mean.

^1^ C: control groups that were exposed to 24°C; HS: heat stress groups that were exposed to 35°C; -Pr: subgroups without dietary propolis supplementation; +Pr: subgroups with dietary propolis supplementation.

^2^ HS: heat stress effect; Pr: propolis effect; HSxPr: interaction between HS and Pr effect. S: significant; NS: non-significant.

### Intestinal histomorphological measurements

The effects of heat stress and dietary propolis supplementation on the histomorphological measurements of quail intestines are presented in Table 3. No significant interactions between HS and Pr treatments were obtained for the histomorphological measurements of quail intestine. However, a significant decrease in the villus width and area was observed due to heat stress. In addition, it was found that dietary propolis supplementation significantly increased the crypts depth of quail intestines.

**Table 3:** Least square means for the histomorphological measurements of small intestines as affected by heat stress and dietary propolis supplementation in Japanese quail.

Items	n	Treatment groups ^1^				SEM	Main effects ^2^		
		C		HS					
		- Pr	+ Pr	- Pr	+ Pr		HS	Pr	HSxPr
Intestinal length (cm)	10	60.0	66.3	63.3	61.7	4.19	NS	NS	NS
Villus length (μm)	10	915.4	940.3	751.9	919.4	72.57	NS	NS	NS
Villus width (μm)	10	192.4 ^a^	155.4 ^b^^c^	128.0 ^c^	149.7 ^b^^c^	15.32	S	NS	NS
Villus area (x10^3^ μm^2^)	10	166.1 ^a^	126.3 ^b^	84.5 ^c^	82.7 ^c^	19.41	S	NS	NS
Crypts depth (μm)	10	80.3 ^b^	171.7 ^a^	109.4 ^a^^b^	131.3 ^a^^b^	19.10	NS	S	NS

^a-c^ Means with different superscripts, within the same row, are significantly different (P<0.05).

n: number of observations per treatment group. SEM: standard error of the mean.

^1^ C: control groups that were exposed to 24°C; HS: heat stress groups that were exposed to 35°C; -Pr: subgroups without dietary propolis supplementation; +Pr: subgroups with dietary propolis supplementation.

^2^ HS: heat stress effect; Pr: propolis effect; HSxPr: interaction between HS and Pr effect. S: significant; NS: non-significant.

**Fig 1:**
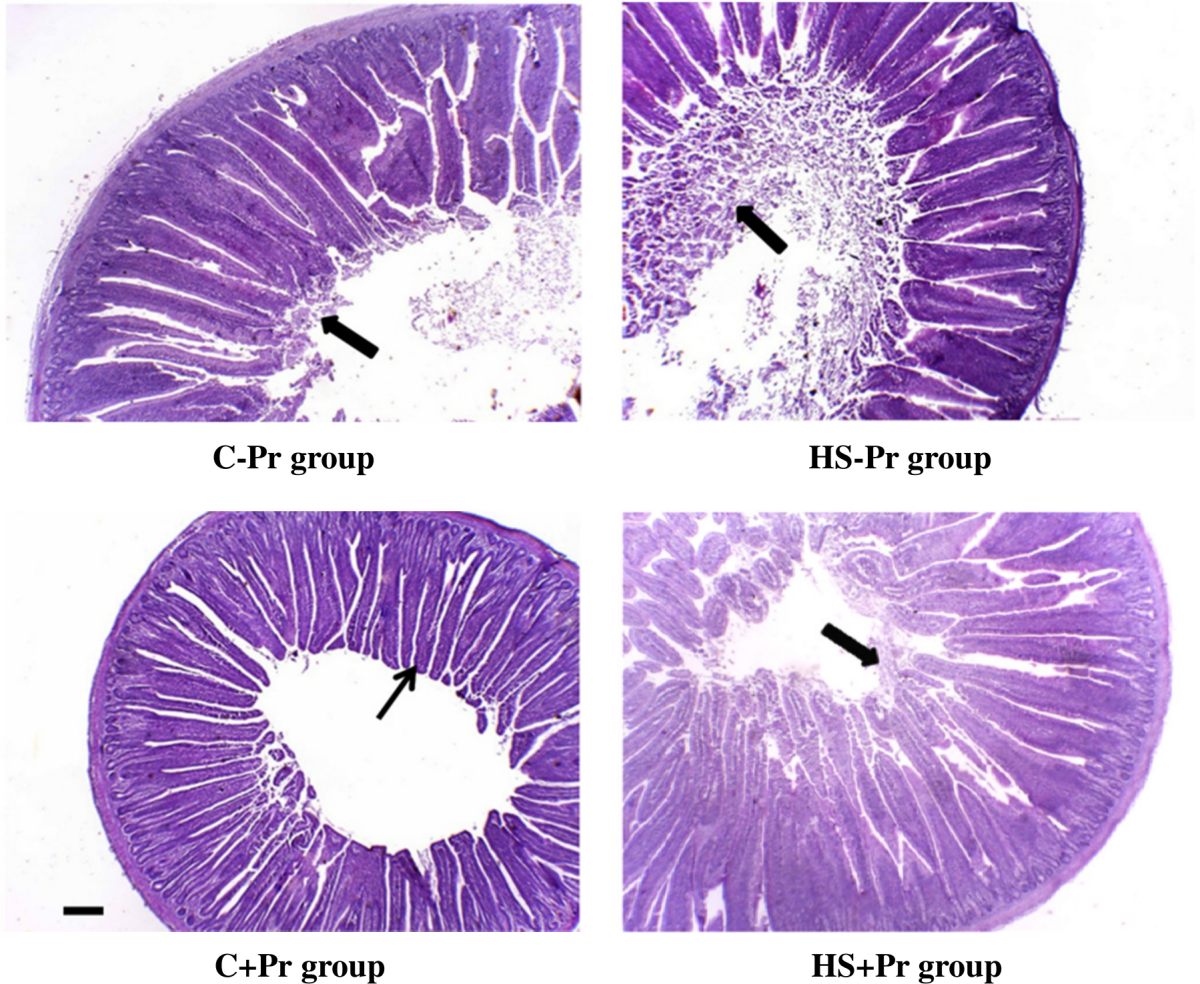
Visual examples for the histomorphological alteration in the intestinal villi of Japanese quail subjected to heat stress and supplemented with propolis in the basal diet (Scale bars 100 μm). C: control groups that were exposed to 24°C; HS: heat stress groups that were exposed to 35°C; -Pr: subgroups without dietary propolis supplementation; +Pr: subgroups with dietary propolis supplementation. The thick arrow indicates the damage and desquamation observed at the tips of the intestinal villi, while the thin arrow indicates the normal structure of intestinal villi.

On the other hand, the histology of quail intestines shows desquamation of the mucosal epithelium and serious disruption of villi due to heat stress treatment. In contrast, dietary propolis supplementation maintains the villus structure relatively normal and alleviates the severe lesions by heat stress (Fig 1).

### Physiological parameters

The effects of heat stress and dietary propolis supplementation on some physiological parameters in Japanese quail are summarized in Table 4. A significant (P<0.05) increase in the body temperature, the plasma corticosterone hormone and the plasma MDA levels was observed in the HS quail group when compared to the C group. While the plasma fT_3_ hormone was significantly lower in the HS group compared to the C group. On the other hand, the body temperature and corticosterone level were significantly decreased while the fT_3_ level was increased due to Pr treatment. Moreover, the high corticosterone level of HS-quail was significantly (P<0.05) reduced by the Pr treatment.

**Table 4:** Least square means for some physiological parameters as affected by heat stress and dietary propolis supplementation in Japanese quail.

Items	n	Treatment groups ^1^				SEM	Main effects ^2^		
		C		HS					
		- Pr	+ Pr	- Pr	+ Pr		HS	Pr	HSxPr
Body temperature (°C)	10	40.5 ^b^^c^	40.2 ^c^	41.4 ^a^	40.7 ^b^	0.15	S	S	NS
Corticosterone (ng/ml)	5	2.8 ^c^	2.6 ^c^	8.0 ^a^	5.0 ^b^	0.56	S	S	S
MDA (nmol/ml)	5	1.8 ^c^	1.8 ^c^	3.4 ^a^	2.4 ^b^	0.24	S	NS	NS
Free T_3_ (pg/ml)	5	4.0 ^a^	5.1 ^a^	2.3 ^b^	4.0 ^a^	0.52	S	S	NS

^a-c^ Means with different superscripts, within the same row, are significantly different (P<0.05).

n: number of observations per treatment group. SEM: standard error of the mean.

^1^ C: control groups that were exposed to 24°C; HS: heat stress groups that were exposed to 35°C; -Pr: subgroups without dietary propolis supplementation; +Pr: subgroups with dietary propolis supplementation.

^2^ HS: heat stress effect; Pr: propolis effect; HSxPr: interaction between HS and Pr effect. S: significant; NS: non-significant.

### Immunological parameters

The results shown in Table 5 indicate that heat stress negatively affected the studied immune response parameters while dietary propolis supplementation enhanced these parameters, in Japanese quail. However, no significant interactions between HS and Pr treatments were obtained for the immunological parameters in the current study.

**Table 5:** Least square means for some immunological parameters as affected by heat stress and dietary propolis supplementation in Japanese quail.

Items	n	Treatment groups ^1^				SEM	Main effects ^2^		
		C		HS					
		- Pr	+ Pr	- Pr	+ Pr		HS	Pr	HSxPr
TWBC’s (x10^3^/mm^3^)	5	81.8 ^c^	131.2 ^a^	60.4 ^d^	108.8 ^b^	8.24	S	S	NS
H/L ratio	5	0.4 ^c^	0.3 ^d^	0.8 ^a^	0.6 ^b^	0.04	S	S	NS
T-lymphocyte proliferation (SI)	5	4.2 ^b^	6.3 ^a^	1.6 ^d^	3.0 ^c^	0.40	S	S	NS

^a-d^ Means with different superscripts, within the same row, are significantly different (P<0.05).

n: number of observations per treatment group. SEM: standard error of the mean.

^1^ C: control groups that were exposed to 24°C; HS: heat stress groups that were exposed to 35°C; -Pr: subgroups without dietary propolis supplementation; +Pr: subgroups with dietary propolis supplementation.

^2^ HS: heat stress effect; Pr: propolis effect; HSxPr: interaction between HS and Pr effect. S: significant; NS: non-significant.

### mRNA quantification

**Fig 2:**
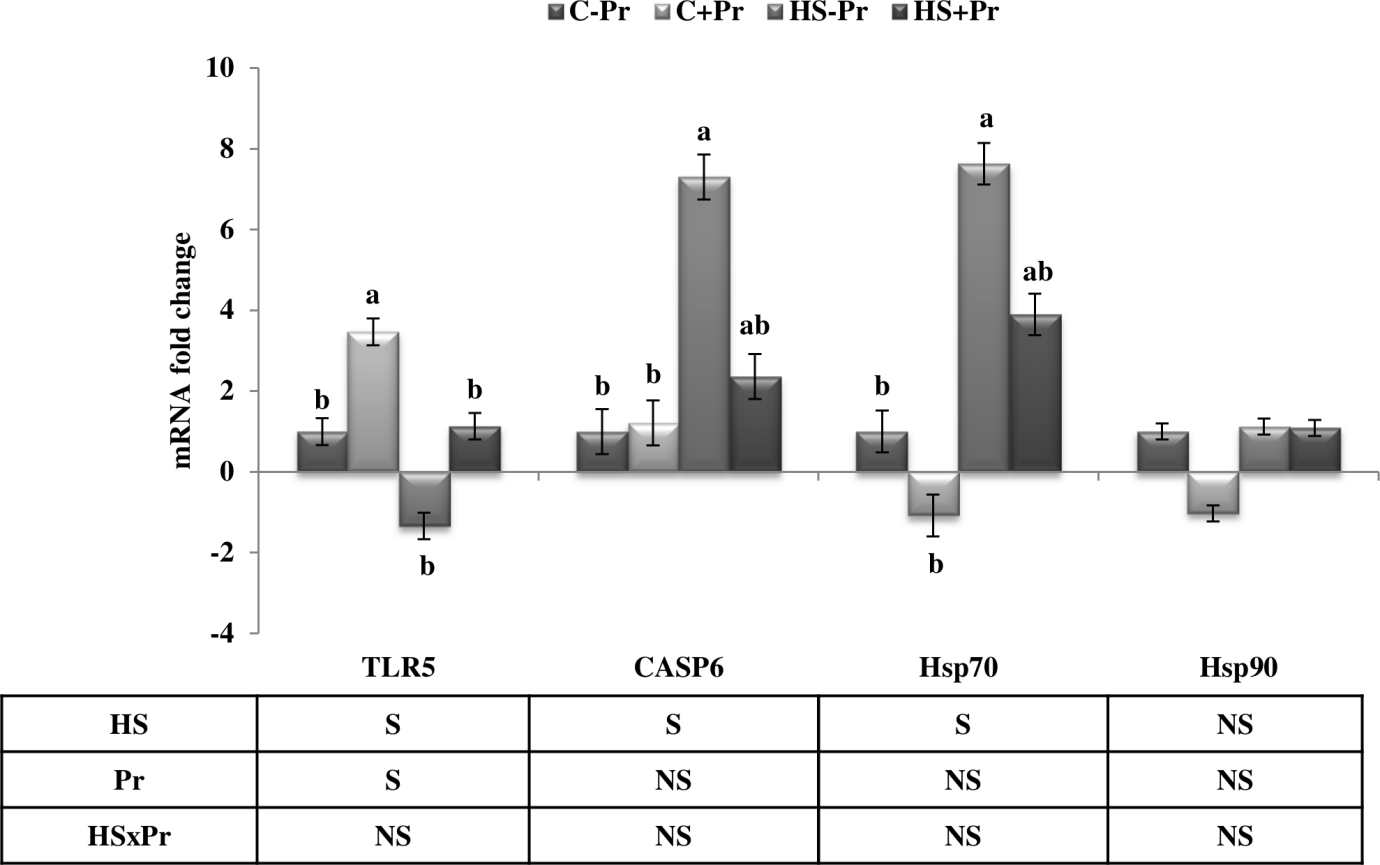
The hepatic relative expression of TLR5, CASP6, Hsp70 and Hsp90 genes as affected by heat stress and dietary propolis supplementation in Japanese quail. Bars express the means ± standard error of means (n = 10). C: control groups that were exposed to 24°C; HS: heat stress groups that were exposed to 35°C; -Pr: subgroups without dietary propolis supplementation; +Pr: subgroups with dietary propolis supplementation. ^a-b^ Means within the same gene with different superscripts are significantly different (P<0.05). The main effects of heat stress (HS), propolis (Pr) and their interaction (HSxPr) are provided for each gene in the table (S: significant; NS: non-significant).

The effects of heat stress and dietary propolis supplementation on the relative expression of examined genes in the liver of Japanese quail are illustrated in Fig 2. There were no significant interactions between HS and Pr treatments for the mRNA expression of selected genes in the present study. However, a significant (P<0.05) augmentation in the expression of TLR5 gene was occurred due to Pr treatment in the C group. In addition, the exposure of quail to heat stress significantly (P<0.05) increased the expression of CASP6 and Hsp70 genes by 7.3 and 7.6 fold, respectively, in the HS-Pr group as compared to the C-Pr group. While propolis decreased the expression of these genes to intermediate levels (2.4 and 3.9 fold for CASP6 and Hsp70, respectively) in the HS+Pr group. No significant differences (P>0.05) were observed in Hsp90 gene expression among the treatment groups.

## Discussion

Global warming became one of the main meteorology factors that may change both the production and the consumption of livestock products in the future. Heat stress has many substantial effects on the biological functions of animals that in turn contribute significantly to the decrease in their productive performance. Exposure of poultry species to heat stress substantially decreases their products which are important and invaluable source of nutrition and livelihood for millions of low income people [39]. It is, therefore, essential to find means to minimize the negative effects of heat stress. Recently, propolis has been used as an alternative and practical way to alleviate the deterioration effects of high environmental temperature in poultry production [12,19]. In the current research, the Japanese quail was used as an experimental animal model to study the capability of dietary propolis supplementation to ameliorate the deleterious effects of heat stress on the production performance, intestinal histomorphology, physiological aspects, immunological responses and the mRNA expression of some immune-stimulatory, apoptotic and heat-shock-protein genes.

In the present study, all production performance traits of the quail were negatively affected by heat stress compared to the control. The low weight gain and feed efficiency were previously reported in broilers [25] and laying hens [20] when they were exposed to heat stress. As indicated by Vona et al. [40], birds decrease their feed intake under heat stress to lower the heat production in their body caused by increased respiration. In addition, the activities of trypsin, chymotrypsin, and amylase become low at a temperature of 32°C [41]. Therefore, high ambient temperatures are associated with lower nutrient digestibility in animals and poultry [42]. On the other hand, the addition of propolis in quail diets significantly increased the weight gain and feed intake, and improved the feed conversion ratio. Similar results were obtained when propolis was added to the quail diets [43] or drinking water [44]. These positive effects of dietary propolis on quail performance could be linked to the palatable substances included in propolis diets like resin, wax, honey and vanillin [45], and these could give beneficial results when birds are not kept at optimal conditions [26].

The changes in intestinal morphology that were observed in the current study could explain the quail production performance under heat stress and effects of dietary propolis supplementation. The villus width and area were significantly lower in the HS group than the C group. Similar results were reported in a previous study on Japanese quail exposed to heat stress [22]. These results could be an outcome of the decreased feed intake in the HS quail, as previously reported in chickens [46]. Consequently, exposure of quail to heat stress may reduce the intact spaces of intestinal epithelia [47], affecting the intestinal digestion and absorption functions [48]; and cause changes that may be sufficient to impair the quail performance as was observed in the current study. No clear improvement was found in the intestinal length and villus measurements after dietary propolis supplementation. Similarly, Denli et al. [43] did not observe changes in the intestinal length of Japanese quail fed with propolis. Such changes may require a longer time of exposure [49]. However, some morphological alterations among the treatment groups were noticed by the visual microscopic examination of villi histology slides. The damage observed in the intestinal villi of the HS-quail groups vs. the integrity of villi structure in the Pr-quail groups indicates that propolis may provide better protection of villi, which is likely to enhance its ability for nutrient absorption [21]. Moreover, crypts depth was significantly deeper in the Pr group. These results are consistent with another study [50], and confirm the positive effect of dietary propolis supplementation on the intestinal morphology and mucosal development. Therefore, the overall histomorphometric changes induced by propolis could be remarkably useful, particularly during heat stress, when the intestinal mucosa is damaged and requires a rapid renewal of villus cells [51].

The data on body temperature from the current research agree with that obtained by Bobek et al. [52] and show that the quail’s body temperature was significantly elevated in the HS group compared to the C group. The failure of thermoregulation process in the HS group may be due to the exergonic reactions of free radicals that are generated by heat stress [53]. In addition, the effects of heat stress on circulating corticosterone and MDA levels in the present study are similar to that obtained in previous works on both broilers and quail [16,54]. These results demonstrate that heat stress induced inflammation and lipid peroxidation of the quail as previously reported [12,55,56]. The excessive amounts of corticosterone may induce the fever that observed in the HS group [16]. Furthermore, the decline in fT_3_ concentrations upon heat exposure is consistent with the reduction in feed consumption of the same quail group in the present study. This might have happened in order to decrease heat accumulation and control body temperature which favors survivability of birds under heat stress condition [57].

It was found that some components extracted from propolis, like flavonoids and caffeic acid phenethyl ester (CAPE), have anti-inflammation capacity [58], anti-oxidant potency [30] and anti-thyroid effects [59], especially under the heat stress environment [54]. In the present study, Pr treatment decreased the plasma corticosterone of the HS quail. The positive effects of propolis partially interfere with oxidative protein denaturation and decrease the protein breakdown [56]. Therefore, the presence of propolis in the quail diets would likely improve the feed conversion ratio and the digestibility of nutrients, reflecting in a high production performance of quail, especially under heat stress conditions.

In line with other works [11,60,61], the current research indicates that heat stress significantly impaired the immunological parameters of Japanese quail. The high H/L ratio and low lymphocyte proliferation observed in the heat-stressed quail may be due to the high plasma corticosterone concentration that observed in the same treatment group [55]. The present study demonstrates that the dietary propolis supplementation significantly improved the immunity parameters in the quail. Other reports suggest that some propolis constituents could augment immune-modulation in the heat-stressed quail via influencing the lymphocyte proliferation and antibody production [62–65]. These compounds also inhibit prostaglandin synthesis as anti-immune substances, thus contribute in getting a better humoral response [66].

To the best of our knowledge, there is no information on the direct effect of propolis at molecular levels in poultry either under thermoneuteral or heat stress conditions. The present research aimed to gain a better understanding of propolis effects following heat stress using qRT-PCR analysis of many genes expressed in the liver of Japanese quail and involved in immunity (TLR5), apoptosis (CASP6) and heat shock proteins (Hsp70 and Hsp90); and at the same time, their expressions have been widely altered in response to stress conditions. The TLR5 gene is a member of the toll-like receptor family which plays a fundamental role in pathogen recognition and activation of innate immunity [67]. While the TLR5 mRNA expression was negatively affected by HS treatment in the current study, the up-regulation effect of propolis on the TLR5 expression leads to the conclusion that supplementing propolis to quail diets could maintain the quail in adequate immune responses, probably by keeping these birds in a well-nourished status [68].

On the other hand, it was observed that the exposure of quail to heat stress significantly increased the expression of CASP6 gene while it was normalized again after propolis supplementation. The CASP6 gene plays a central role in the execution-phase of cell apoptosis [69], and could be up-regulated as a result of the increased fever, corticosterone and MDA levels in the heat-stressed quail [16]. The protective effects of propolis and its components against the activation of the caspase cascade that leads to apoptosis and subsequent cell death were also reported in previous works on human cells [29,70]. Furthermore, two classes of heat shock proteins; Hsp70 and Hsp90, which are mainly synthesized to protect cells when stressed by elevated temperatures [71]; were also analyzed in the present study. The up-regulation of hepatic Hsp70 expression in the heat-stressed quail may be promoted in a consequence of the elevated plasma levels of MDA in these quail in order to protect cells against peroxidation damage [72]. The protective effect of Hsp70 does not correlate with the intestinal morphology under heat stress, but it may be strongly correlated with the increased activity of the digestive enzymes [49]. Moreover, many signaling pathways correlated with apoptosis and protein transcription are modulated by Hsp90 to improve the tolerance to stress [73]. The study of Xie et al. [15] indicated that mRNA expression of Hsp90 in the liver of broiler breeder hens increased after a long-term heat treatment. However, the current study does not show any differences in the hepatic Hsp90 gene expression in the quail groups exposed to heat stress when compared to the control group.

## Conclusion

The results of the present study indicate that dietary propolis supplementation can alleviate the negative effects of heat stress on the growth performance, intestinal histomorphometry, physiology and immunity of Japanese quail. Propolis maintained the normal levels of plasma free T_3_ hormone, preserved the integrity of intestinal villi and increased the crypts depth in the heat-stressed quail, which consequently enhanced the feed efficiency and growth performance of the quail. The plasma corticosterone and body temperature were decreased in the quail that were fed with propolis, indicating that propolis decreases the stress indicators in quail exposed to heat stress conditions. Moreover, the quail that were fed with propolis expressed good immunity status as presented by a lower H/L ratio and higher lymphocyte proliferation together with the high expression of TLR5 gene. Furthermore, the positive effects of propolis were evidenced by normalizing the high expressions of CASP6-apoptotic gene and Hsp70-stress gene to lower levels in the heat-stressed quail. Therefore, the addition of propolis at a rate of 1 g/kg to the diet of quail could be recommended as a potential nutritional strategy in order to improve their performance, especially under heat stress conditions.

## Supporting information

S1 Checklist The ARRIVE (animal research: Reporting of in vivo experiments) guidelines checklist. (PDF)
